# Ethanol Enhances High-Salinity Stress Tolerance by Detoxifying Reactive Oxygen Species in *Arabidopsis thaliana* and Rice

**DOI:** 10.3389/fpls.2017.01001

**Published:** 2017-07-03

**Authors:** Huong Mai Nguyen, Kaori Sako, Akihiro Matsui, Yuya Suzuki, Mohammad Golam Mostofa, Chien Van Ha, Maho Tanaka, Lam-Son Phan Tran, Yoshiki Habu, Motoaki Seki

**Affiliations:** ^1^Plant Genomic Network Research Team, RIKEN Center for Sustainable Resource Science (CSRS)Yokohama, Japan; ^2^Kihara Institute for Biological Research, Yokohama City UniversityYokohama, Japan; ^3^Core Research for Evolutional Science and Technology, Japan Science and Technology AgencyKawaguchi, Japan; ^4^Institute of Agrobiological Sciences, National Agriculture and Food Research OrganizationTsukuba, Japan; ^5^Signaling Pathway Research Unit, RIKEN Center for Sustainable Resource Science (CSRS)Yokohama, Japan

**Keywords:** salinity stress, ethanol, organic solvent, reactive oxygen species, rice

## Abstract

High-salinity stress considerably affects plant growth and crop yield. Thus, developing techniques to enhance high-salinity stress tolerance in plants is important. In this study, we revealed that ethanol enhances high-salinity stress tolerance in *Arabidopsis thaliana* and rice. To elucidate the molecular mechanism underlying the ethanol-induced tolerance, we performed microarray analyses using *A. thaliana* seedlings. Our data indicated that the expression levels of 1,323 and 1,293 genes were upregulated by ethanol in the presence and absence of NaCl, respectively. The expression of reactive oxygen species (ROS) signaling-related genes associated with high-salinity tolerance was upregulated by ethanol under salt stress condition. Some of these genes encode ROS scavengers and transcription factors (e.g., *AtZAT10* and *AtZAT12*). A RT-qPCR analysis confirmed that the expression levels of *AtZAT10* and *AtZAT12* as well as *AtAPX1* and *AtAPX2*, which encode cytosolic ascorbate peroxidases (APX), were higher in ethanol-treated plants than in untreated control plants, when exposure to high-salinity stress. Additionally, *A. thaliana* cytosolic APX activity increased by ethanol in response to salinity stress. Moreover, histochemical analyses with 3,3′-diaminobenzidine (DAB) and nitro blue tetrazolium (NBT) revealed that ROS accumulation was inhibited by ethanol under salt stress condition in *A. thaliana* and rice, in which DAB staining data was further confirmed by Hydrogen peroxide (H_2_O_2_) content. These results suggest that ethanol enhances high-salinity stress tolerance by detoxifying ROS. Our findings may have implications for improving salt-stress tolerance of agriculturally important field-grown crops.

## Introduction

High-salinity stress is detrimental to plant growth and productivity, and causes considerable yield losses to economically important crops, thereby threatening sustainable agriculture (Shrivastava and Kumar, 2015). Thus, it is essential that methods to enhance high-salinity stress tolerance are developed. A recent study summarized that certain chemical compounds can be used to enhance plant stress tolerance (Savvides et al., 2016). Other studies confirmed that the application of exogenous chemical compounds enhance high-salinity stress tolerance in many plant species. These chemicals included phytohormones such as salicylic acid, methyl jasmonate, and strigolactone (Yoon et al., 2009; Ha et al., 2014; Khan et al., 2015). Epigenetic inhibitors, such as Ky-2 and suberoylanilide hydroxamic acid (Sako et al., 2016; Patanun et al., 2017), and other chemical compounds, including sodium nitroprusside, melatonin, and polyamines (Savvides et al., 2016), can also improve tolerance to salt stress condition.

One of the molecular effects of chemical compounds that enhance abiotic stress tolerance in plants involves the activation of antioxidant processes. Reactive oxygen species (ROS) are toxic to proteins, lipids, carbohydrates, and DNA, and ultimately lead to membrane damage and cell death (Gill and Tuteja, 2010). ROS generated by NADPH oxidase accumulate under stress conditions leading to the production of singlet oxygen (^1^O_2_) and a superoxide anion radical (${\text{O}}_{2}^{•-}$), which are converted to H_2_O_2_. The H_2_O_2_ is then converted to a hydroxyl radical (HO^•^) via the metal-dependent Haber-Weiss reaction or the Fenton reaction. The excess HO^•^ can react with lipids and results in the degradation of the cell membrane, which is an important barrier that protects plant cells (Asada, 2006). ROS homeostasis is regulated by the antagonism between ROS producers and scavengers. Several reports have described the network of ROS signaling genes in *Arabidopsis thaliana* (Mittler et al., 2004; Gadjev et al., 2006; Miller et al., 2010). Thus, the induction of genes encoding for key enzymes that regulate ROS accumulation, such as superoxide dismutases (SODs), ascorbate peroxidases (APXs), catalases (CATs), and other peroxidases, is necessary to remove excess ${\text{O}}_{2}^{•-}$ and H_2_O_2_ and ensure plant survival (Miller et al., 2010). Additionally, in *A. thaliana*, the expression of antioxidant defense genes is regulated by related transcription factors, including *AtZAT10* and *AtZAT12* (Rizhsky et al., 2004; Mittler et al., 2006; Miller et al., 2008).

Organic solvents, such as acetone, dimethyl sulfoxide (DMSO), *N,N-*dimethylformamide (DMF), ethanol, and methanol, are commonly used to dissolve compounds during experiments (Savvides et al., 2016). However, their effects on plant stress responses and tolerance have not been elucidated. Ethanol is a volatile, flammable, and colorless liquid, with a slight odor. Ethanol fermentation is one of the fundamental processes occurring during plant stress responses, and is necessary for responses to low-oxygen stress conditions (Tadege et al., 1999). Endogenous ethanol is produced under anaerobic conditions as part of a fermentation pathway (Kimmerer and Kozlowski, 1982; Kimmerer and MacDonald, 1987). Although, rice plants treated with exogenous ethanol have been reported to exhibit tolerance to chilling stress (Kato-Noguchi, 2008), it is unclear whether an ethanol treatment can enhance high-salinity stress tolerance in plants.

We herein provide new insights into the biological functions of ethanol influencing plant responses and tolerance to high-salinity stress. We revealed that the application of exogenous ethanol enhances high-salinity stress tolerance by regulating ROS-related genes and enhancing ROS detoxification.

## Materials and methods

### Plant materials and growth conditions

*A. thaliana* (ecotype Columbia-0) seeds were sterilized and sown in half-strength Murashige and Skoog (MS) liquid medium supplemented with 1% sucrose and 0.1% agar. The plants were grown under previously described conditions (Sako et al., 2016). Four-day-old plants were treated with ethanol (Wako, Japan), acetone (Wako, Japan), methanol (Wako, Japan), *N'N-*dimethylformamide (DMF) (Wako, Japan), dimethyl sulfoxide (DMSO) (Wako, Japan), or sterilized deionized water for 24 h, with or without a subsequent treatment with 100 mM NaCl (Wako, Japan). The NaCl solution was added into the medium containing the solvents (Figure S1A). The survival rate of 20 plants was calculated 4 days after the NaCl treatment. The experiment was conducted using three biological replicates.

For rice experiments, *Oryza sativa* L. cv. Nipponbare seeds were germinated in water at 30°C for 2 days, and then transferred to plastic pots containing granular soil (Bonsoru No. 2; Sumitomo Chemical, Tokyo). The plants were grown in a vat filled with water at 30°C for 2 weeks under a 14-h light:10-h dark photoperiod. For the salinity stress test, soil moisture was removed by leaving the pots on Kimtowel (Nippon Paper Crecia) for 20 min. The pots were then incubated in a 0, 0.3, or 0.6% (corresponding to 0, 51 or 103 mM, respectively) ethanol solution for 4 days. After the ethanol treatment, the pots were left on Kim towel for 20 min to remove soil moisture. The pots were subsequently transferred to a 200 mM NaCl solution and incubated for 5 days. The data was analyzed from 12 plants for each treatment. The experiment was conducted using two independent biological replicates.

### Measurement of chlorophyll content

Four-day-old *A. thaliana* plants were treated with 0.3% (51 mM) ethanol for 24 h and then exposed to 100 mM NaCl for 72 h. We then measured the chlorophyll content of 30–50 mg seedlings for each treatment as previously described (Kim et al., 2003). The experiment was conducted with three biological replicates. Statistical significance was determined by ANOVA, followed by *post-hoc* Tukey's tests. Means that differed significantly (*P* < 0.05) are indicated by different letters.

### RNA extraction

Total RNA was extracted from 5-day-old *A. thaliana* seedlings that were treated with 0.3% (51 mM) ethanol for 24 h, with or without a subsequent treatment with 100 mM NaCl for 2 h. Sterilized deionized water was used as a negative control. The RNA was extracted using the Plant RNA reagent (Thermo Fisher Scientific) as previously described (Nguyen et al., 2015). The quality of the extracted total RNA was evaluated using a Bioanalyzer system (Agilent). The RNA was extracted from 30 plants. The experiment was conducted using three biological replicates.

### Microarray analysis

A microarray analysis was completed as previously described (Nguyen et al., 2015). The microarray data underwent a one-way ANOVA method and were deposited in the GEO database (GEO ID: GSE95202). Each treatment was analyzed using four biological replicates. A total of 30 plants were used for each treatment. Genes with an expression log_2_ ratio ≥ 0.7 [*t*-test analysis, Benjamini–Hochberg correction (FDR) ≤ 0.05] were identified as upregulated genes.

### Quantitative real-time PCR analysis

We synthesized cDNA using the QuantiTect Reverse Transcription Kit (QIAGEN) for a quantitative real-time polymerase chain reaction (qRT-PCR) analysis. The qRT-PCR was conducted as previously described (Sako et al., 2016). We used *AtACT2* as a reference gene. The experiment was conducted using three biological replicates. A total of 30 plants were used for each treatment. The qRT-PCR primer sequences were as follows: *AtZAT10*: 5′-ACATCCCTCCGATCCCTGAA-3′ and 5′-ACCGGAAAGTCAAACCGAGG-3′; *AtZAT12*: 5′-TCCGATGGGACAAGCTTTGG-3′ and 5′-AAGCCACTCTCTTCCCACTG-3′; *AtAPX1*: 5′-GCACTATTGGACGACCCTGT-3′ and 5′-AGCAAACCCAAGCTCAGAAA-3′; *AtAPX2*: 5′-AAGTTGAGCCACCTCCTGAA-3′ and 5′-GTGTGTCCACCAGACAATGC-3′; *AtACT2*: 5′-GATCTCCAAGGCCGAGTATGAT-3′ and 5′-aCCCATTCATAAAACCCCAGC-3′.

### Ascorbate peroxidase assay

Five-day-old *A. thaliana* plants treated with 0.3% (51 mM) ethanol for 24 h, with or without a subsequent treatment of 100 mM NaCl for 12 h were used for an APX assay. The experiment was conducted using three biological replicates. Proteins were extracted from 30 plants and the APX assay was conducted as previously described (Bradford, 1976; Mostofa et al., 2015). The protein content was determined using bovine serum albumin as a standard.

### Staining to detect the superoxide anion and hydrogen peroxide

Five-day-old *A. thaliana* plants treated with 0.3% (51 mM) ethanol for 24 h, with or without a subsequent treatment with 100 mM NaCl for 12 h were stained using a modified version of a published method (Kumar et al., 2014; Mostofa et al., 2015). To detect ${\text{O}}_{2}^{•-}$, plants were stained for 30 min with 0.05% NBT (w/v) in 50 mM potassium phosphate, pH 7.0. To detect H_2_O_2_, plants were stained for 5 h with 0.1% DAB in 10 mM potassium phosphate, pH 7.0. Samples were stained under light at room temperature, after which they were cleared with an ethanol:acetic acid (96:4) solution until photographed by a digital microscope (VHX-5000, Keyence). The experiment was conducted using three biological replicates. A total of 30 plants were used for each treatment.

For the rice experiments, 14-day-old *O. sativa* L. cv. Nipponbare plants treated with or without 0.3% (51 mM) ethanol for 24 h were exposed to 100 mM NaCl for 24 h. The second leaf was stained with NBT or DAB as previously described (Mostofa et al., 2015) and then photographed using the M165 FC fluorescent stereo microscope (Leica).

### Measurement of hydrogen peroxide

Four-day-old *A. thaliana* plants were treated with 0.3% (51 mM) ethanol for 24 h and then exposed to 100 mM NaCl for 72 h. H_2_O_2_ content was then measured as described previously (Ivanchenko et al., 2013). The experiment was conducted with three biological replicates.

For the rice experiments, 14-day-old *O. sativa* L. cv. Nipponbare plants were treated with or without 0.3% (51 mM) ethanol for 24 h and then exposed to 100 mM NaCl for 24 h. The second leaf was sampled for H_2_O_2_ content as described previously (Ivanchenko et al., 2013). The experiment was conducted with three biological replicates.

## Results

### Ethanol enhances high-salinity stress tolerance in *Arabidopsis thaliana*

**Figure 1:**
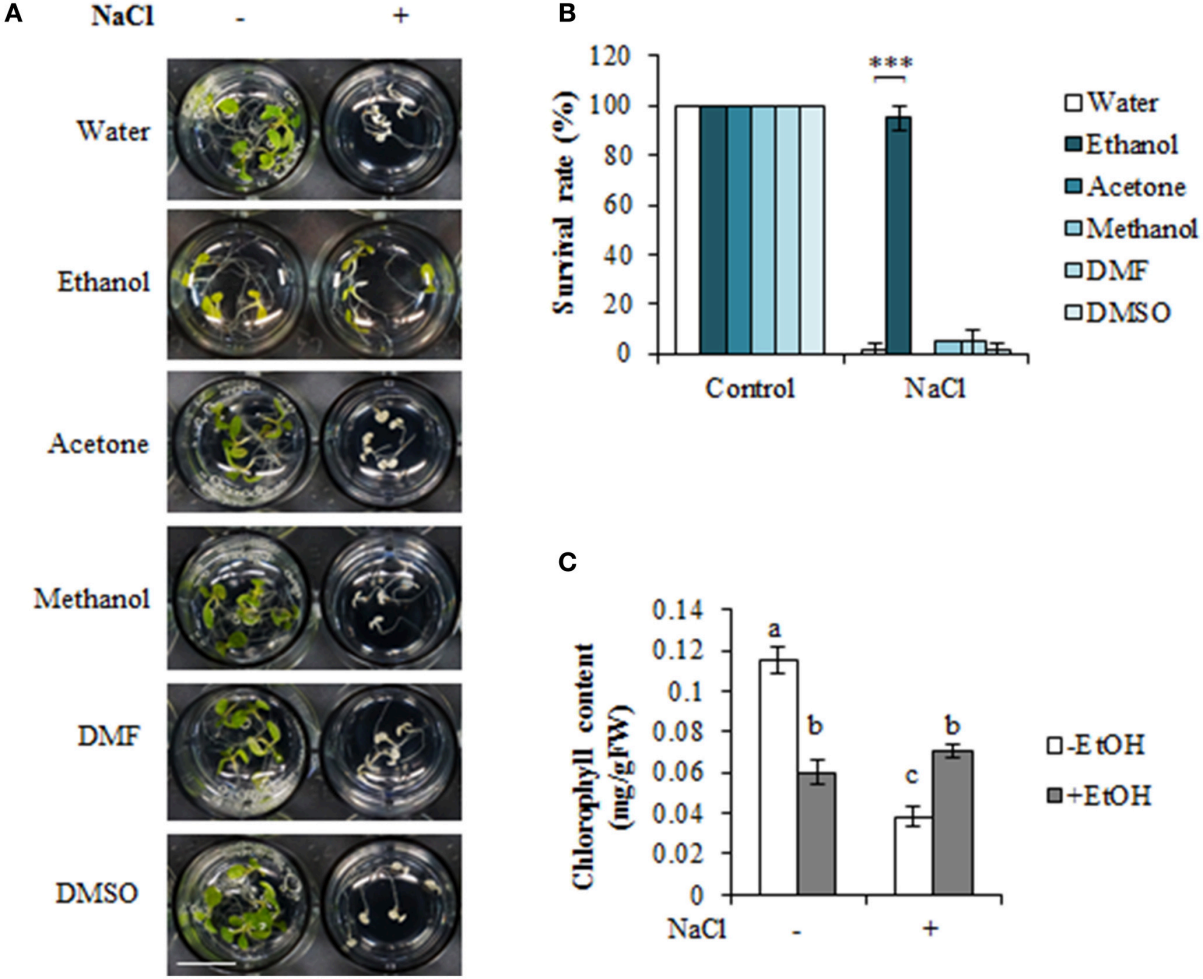
Ethanol enhances high-salinity stress tolerance in *Arabidopsis thaliana*. **(A)** Phenotype of *A. thaliana* seedlings treated with 0.3% (51 mM) organic solvent, with or without a subsequent treatment with 100 mM NaCl for 4 days. Water was used as a negative control. Bars = 1 cm. **(B)** Survival rate under high-salinity condition in the presence or absence of various organic solvents. The survival rate of 20 plants was calculated on 4 days after the NaCl treatment. The experiment was conducted using three biological replicates. Error bars represent the mean ± standard deviation (*SD*). Significance was determined according to Student's *t*-test. ^***^P < 0.00001. **(C)** Chlorophyll content in 0.3% (51 mM) ethanol-treated and untreated plants under high-salinity condition. Error bars represent the mean ± *SD*, three independent biological repeats were performed. Statistical significance was determined by ANOVA, followed by *post-hoc* Tukey's tests. Means that differed significantly (*P* < 0.05) are indicated by different letters.

We examined the effects of five organic solvents on *A. thaliana* high-salinity stress tolerance (Figures 1A,B, Figure S1A). Wild-type plants grown in liquid culture medium were treated with an organic solvent or water for 24 h, with or without a subsequent treatment with 100 mM NaCl for 4 days (Figure S1A). We observed that the ethanol treatment enhanced *A. thaliana* high-salinity stress tolerance (Figures 1A,B), although the plants appeared slightly yellow under the non-stress condition. In contrast, plants treated with acetone, methanol, DMF, or DMSO did not exhibit any significant morphological differences compared with the control plants under the non-stress condition. Additionally, plants were unable to survive under the high-salinity stress condition (Figures 1A,B). The effects of various concentrations of organic solvents were also tested as shown in Figure S1. The data showed that the other organic solvents except for ethanol could not rescue plants from high-salinity stress condition. Consistent with these results, we observed that the chlorophyll content was higher in ethanol-treated plants than in the untreated plants under the high-salinity stress condition (Figure 1C).

Because the ethanol-treated plants became slightly yellow, we also evaluated the survival rate of salinity-stressed plants after a 7-day recovery period, during which the plants were transferred to MS liquid medium (Figure S2). A higher recovery rate was observed for ethanol-treated plants (93%) than for untreated seedlings (50%; Figure S2). Our data confirmed that ethanol enhances *A. thaliana* tolerance to high-salinity stress.

### Microarray-based identification of candidate genes associated with ethanol-mediated high-salinity tolerance

**Figure 2:**
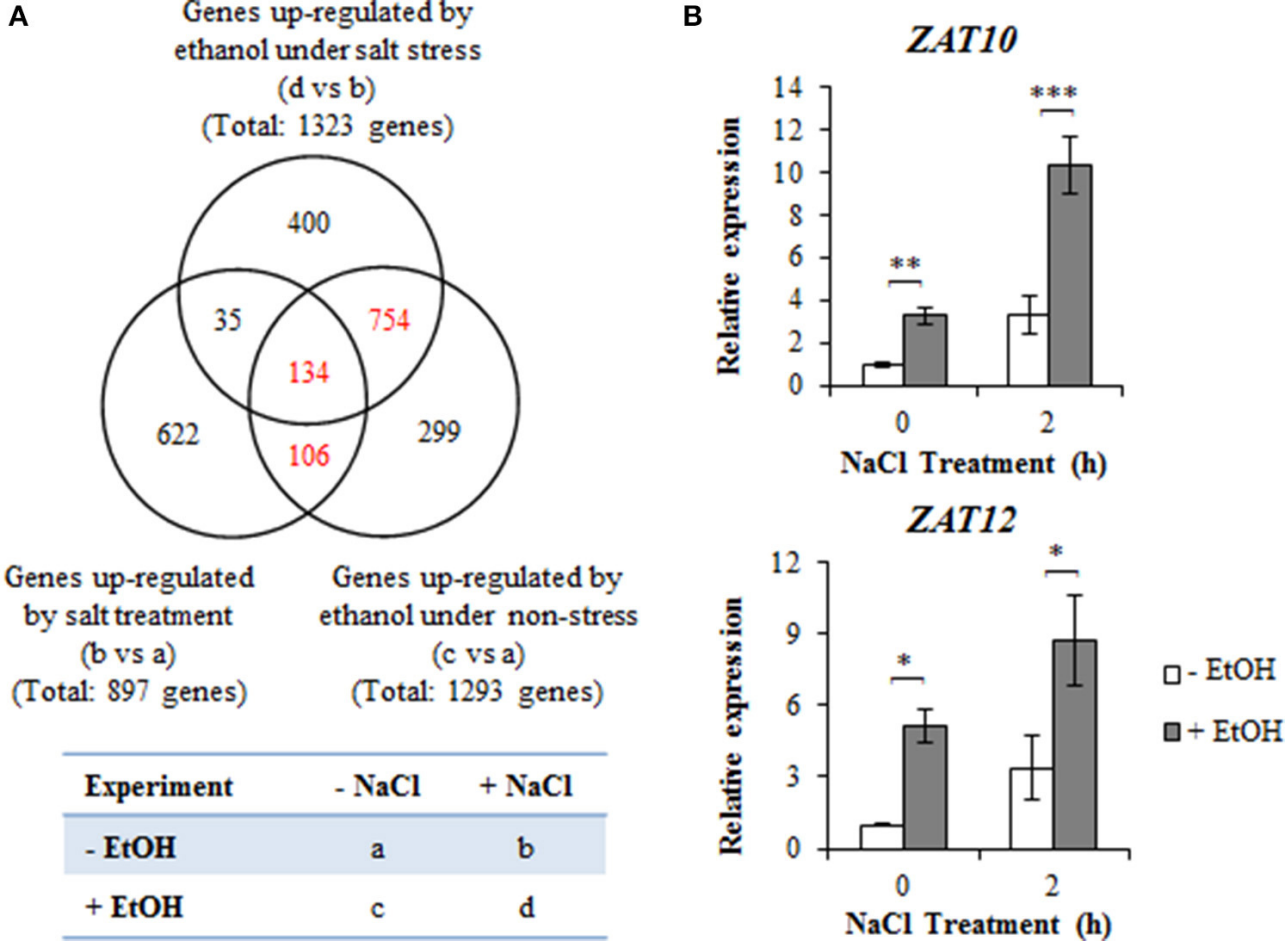
Expression profiles of genes upregulated by ethanol and high-salinity stress treatments. **(A)** Venn diagram with 1,323 genes (d vs. b) upregulated in ethanol-treated plants under high-salinity condition, 1,293 genes (c vs. a) upregulated in ethanol-treated plants in the absence of salinity stress, and 897 genes (b vs. a) upregulated in salt-stressed plants in the absence of an ethanol treatment. Each treatment was analyzed using 30 plants. Four biological repeats were performed. **(B)** Relative *AtZAT10* and *AtZAT12* expression levels during a salinity stress treatment for 0 and 2 h in the presence or absence of 0.3% (51 mM) ethanol. The expression level of the unstressed plants treated with water was set as 1, and the *ACT2* gene was used as an internal standard. Each treatment was analyzed using 30 plants. Three biological repeats were performed. Error bars represent the mean ± *SD*. Significance was determined according to Student's *t*-test. ^*^P < 0.05; ^**^P < 0.01; ^***^P < 0.001.

We analyzed the ethanol-induced gene expression levels associated with *A. thaliana* high-salinity stress tolerance using a microarray. Four-day-old plants treated with 0.3% (51 mM) ethanol or water for 24 h, with or without a subsequent treatment with 100 mM NaCl for 2 h were examined (Figure 2A). We observed that 1,293 genes were more highly expressed in ethanol-treated plants than in the untreated control plants in the absence of high-salinity stress (Figure 2A, Table S1). Among these genes, 240 exhibited upregulated expression following a 2 h NaCl treatment in the absence of ethanol (Figure 2A, Table S2). We observed that 1,323 genes were more highly expressed in plants treated with NaCl in the presence of ethanol than in plants treated with NaCl in the absence of ethanol (Figure 2A, Table S3). Of these genes, 169 overlapped with salinity stress-upregulated genes (Figure 2A, Table S4), while 888 genes overlapped with ethanol-upregulated genes in the absence of NaCl (Figure 2A, Table S5). Furthermore, we detected 134 NaCl-inducible genes that were more highly expressed in ethanol-treated plants than in untreated controls, with or without NaCl treatment (Figure 2A, Table S6).

For a more detailed analysis, we focused on the 994 overlapping genes highlighted in red in the Venn diagram presented in Figure 2A. Of these genes, 35 were related to ROS signaling (Table 1), including genes encoding ROS-scavengers [e.g., APX, CAT, glutathione peroxidase (GPX), peroxiredoxin (PrxR), glutathione S-transferase (GST), and alternative oxidase (AOX)], ROS-scavenging signaling molecules (e.g., ferritin and blue copper proteins, which inhibit the production of HO^•^, glutathione reductase, dehydroascorbate reductase, and glutaredoxin). In contrast, the expression of *SOD* genes encoding ${\text{O}}_{2}^{•-}$ scavengers was unaffected by ethanol under high-salinity condition.

**Table 1:** List of ROS signaling-related genes^a^ that were up-regulated by both ethanol and NaCl.

**Gene name**	**AGI code**	**Salt/control**			**Ethanol/control under non-stress**			**Ethanol/control under salt stress**		
		**Ratio^b^**	***p*-value**	**FDR**	**Ratio^c^**	***p*-value**	**FDR**	**Ratio^d^**	***p*-value**	**FDR**
*APX2; Ascobate Peroxidase 2*	*AT3G09640*	−0.704	2.2E-03	2.3E-02	1.248	1.3E-03	9.8E-03	0.953	9.6E-04	6.6E-03
*CAT2; Catalase 2*	*AT4G35090*	0.049	6.0E-01	8.1E-01	0.710	2.1E-04	2.6E-03	0.837	1.0E-04	1.3E-03
*GPX7; Glutathione Peroxidase 7*	*AT4G31870*	−0.009	9.7E-01	9.9E-01	0.942	1.9E-04	2.4E-03	1.087	1.3E-03	8.5E-03
*GPX6; Phospholipid Glutathione Peroxidase 6*	*AT4G11600*	1.013	1.3E-06	3.2E-04	0.707	1.4E-04	2.0E-03	0.556	4.3E-05	7.4E-04
*AOX1A; Alternative oxidase 1A*	*AT3G22370*	3.126	1.2E-07	1.1E-04	4.217	4.1E-08	3.2E-05	1.403	7.3E-07	7.6E-05
*Type 2 PrxR D; Type 2 Peroxiredoxin D*	*AT1G60740*	−0.174	2.5E-01	5.3E-01	1.423	1.6E-06	1.5E-04	1.483	2.0E-05	4.5E-04
*GST7; glutathione S-transferase 7*	*AT1G02920*	0.463	4.1E-03	3.6E-02	1.223	1.1E-04	1.7E-03	1.047	3.7E-06	1.7E-04
*GST6; glutathione S-transferase 6*	*AT1G02930*	−0.175	2.5E-01	5.3E-01	0.535	1.1E-03	8.3E-03	0.793	1.2E-03	7.9E-03
*GSTU1; glutathione S-transferase TAU 1*	*AT2G29490*	0.573	3.9E-05	1.8E-03	3.023	4.9E-07	9.1E-05	2.476	1.5E-08	2.6E-05
*GSTU2; glutathione S-transferase tau 2*	*AT2G29480*	0.670	8.8E-04	1.2E-02	1.598	2.2E-05	5.9E-04	1.828	1.3E-06	1.0E-04
*GSTU4; glutathione S-transferase tau 4*	*AT2G29460*	1.067	4.1E-02	1.7E-01	2.273	1.3E-05	4.4E-04	1.869	3.8E-03	1.9E-02
*GSTU7; glutathione S-transferase tau 7*	*AT2G29420*	0.291	2.7E-02	1.3E-01	2.291	6.1E-07	9.7E-05	2.073	8.3E-07	8.3E-05
*GSTU8; glutathione S-transferase TAU 8*	*AT3G09270*	0.615	1.1E-04	3.3E-03	1.813	4.3E-07	8.8E-05	1.560	5.4E-07	6.7E-05
*GSTU9; glutathione S-transferase tau 9*	*AT5G62480*	2.764	2.4E-07	1.5E-04	3.384	9.9E-08	4.7E-05	1.099	1.0E-05	3.0E-04
*GSTU12; glutathione S-transferase TAU 12*	*AT1G69920*	1.242	6.9E-03	5.1E-02	1.433	1.3E-03	9.8E-03	1.878	4.2E-04	3.6E-03
*GSTU19; glutathione S-transferase TAU 19*	*AT1G78380*	−0.023	7.7E-01	9.0E-01	1.450	1.3E-07	5.2E-05	1.422	3.5E-07	5.6E-05
*GSTU16; glutathione S-transferase TAU 16*	*AT1G59700*	0.668	5.1E-04	8.7E-03	1.204	1.9E-07	6.5E-05	0.942	5.4E-05	8.6E-04
*GSTU22; glutathione S-transferase TAU 22*	*AT1G78340*	2.416	1.7E-06	3.6E-04	4.276	2.1E-08	2.6E-05	2.720	2.1E-07	4.7E-05
*GSTU24; glutathione S-transferase TAU 24*	*AT1G17170*	−0.730	9.8E-03	6.6E-02	3.822	5.7E-06	2.8E-04	4.095	4.7E-09	1.9E-05
*GSTU25; glutathione S-transferase TAU 25*	*AT1G17180*	0.632	6.8E-04	1.0E-02	3.851	9.8E-09	1.9E-05	3.421	2.7E-08	2.9E-05
*GSTF8; glutathione S-transferase phi 8*	*AT2G47730*	−0.142	1.2E-01	3.5E-01	1.100	3.9E-05	8.6E-04	0.983	1.9E-05	4.3E-04
*GSTF12; glutathione S-transferase phi 12*	*AT5G17220*	−0.031	9.1E-01	9.7E-01	1.629	1.2E-06	1.3E-04	1.680	6.6E-04	5.0E-03
*GSTZ1; glutathione S-transferase zeta 1*	*AT2G02390*	0.096	1.6E-01	4.1E-01	1.306	7.5E-06	3.2E-04	1.217	4.7E-07	6.4E-05
*Glutathione S-transferase family protein*	*AT1G10370*	0.129	2.2E-01	4.9E-01	1.094	1.3E-06	1.4E-04	0.937	6.7E-05	1.0E-03
*microsomal glutathione s-transferase, putative*	*AT1G65820*	0.014	7.4E-01	8.9E-01	0.881	1.1E-06	1.3E-04	0.881	8.2E-08	3.5E-05
*Glutathione S-transferase family protein*	*AT4G19880*	0.237	3.1E-02	1.4E-01	1.761	4.8E-08	3.3E-05	1.498	3.2E-06	1.6E-04
*DHAR3; Dehydroascorbate Reductase 3*	*AT1G75270*	−0.157	8.5E-02	2.8E-01	1.715	3.3E-07	8.2E-05	1.629	5.5E-07	6.8E-05
*GR1; Glutathione Reductase 1*	*AT3G24170*	−0.105	4.2E-03	3.7E-02	0.738	3.0E-05	7.2E-04	0.777	6.1E-08	3.1E-05
*Ferritin 1*	*AT5G01600*	−0.050	6.4E-01	8.4E-01	1.261	1.1E-04	1.6E-03	1.100	1.5E-05	3.8E-04
*Ferritin 4*	*AT3G11050*	−0.197	1.9E-01	4.6E-01	1.150	3.1E-05	7.4E-04	1.201	1.8E-04	1.9E-03
*blue copper protein, putative*	*AT3G27200*	0.101	5.1E-01	7.6E-01	1.012	2.5E-04	2.9E-03	0.723	1.1E-03	7.5E-03
*glutaredoxin family*	*AT1G28480*	2.813	8.2E-04	1.2E-02	1.368	5.1E-04	4.9E-03	0.757	2.2E-01	4.2E-01
*glutaredoxin family*	*AT3G62960*	−0.651	5.8E-03	4.5E-02	0.844	3.2E-04	3.4E-03	0.560	9.0E-03	3.7E-02
*glutaredoxin family*	*AT4G33040*	0.101	2.9E-01	5.8E-01	0.953	1.0E-04	1.6E-03	1.523	2.1E-07	4.7E-05
*glutaredoxin family*	*AT5G11930*	−0.560	4.0E-05	1.8E-03	0.770	3.4E-05	7.7E-04	1.041	5.5E-06	2.1E-04

a *The genes with the following at least two categories are listed: (1) log_2_ ratio (plants treated with water for 24 h followed by 2 h NaCl treatment/plants treated with water for 24 h) ≥ 0.7, FDR ≤ 0.05, t-test ≤ 0.05; (2) log_2_ ratio (plants treated with ethanol for 24 h/plants treated with SDW for 24 h) ≥ 0.7, FDR ≤ 0.05, t-test ≤ 0.05; (3) log_2_ ratio (plants treated with NaCl in the presence of ethanol/plants treated with NaCl in the absence of ethanol) ≥ 0.7, FDR ≤ 0.05, t-test ≤ 0.05*.

b *The values represent the log_2_ ratio (plants treated with water for 24 h followed by 2 h NaCl treatment/plants treated with water for 24 h)*.

c *The values represent the log_2_ ratio (plants treated with ethanol for 24 h/plants treated with water for 24 h)*.

d *The values represent the log_2_ ratio (plants treated with NaCl in the presence of ethanol / plants treated with NaCl in the absence of ethanol)*.

The expression of several transcription factor family genes involved in ROS signaling was also induced by ethanol in salt-stressed plants. These genes encoded C_2_H_2_ zinc finger proteins (*AtZAT6_AT5G04340, AtZAT10_AT1G27730*, and *AtZAT12_AT5G59820*), WRKY proteins (*AtWRKY6_AT1G62300, AtWRKY25_AT2G30250*, and *AtWRKY33_AT2G38470*), a DREB protein (*AtDREB19_AT2G38340*), a heat shock factor protein (*AtHsfA4A_AT4G18880*), and NAC proteins (*ANAC019_AT1G52890, ANAC102_ AT5G63790*, and *ANAC032_AT1G77450*) (Tables S2, S5, S6). The *AtZAT10* and *AtZAT12* transcription factor genes were further analyzed by qRT-PCR. The expression levels of these two genes increased following ethanol and NaCl treatments (Figure 2B). These observations suggest that the salt tolerance conferred by ethanol might be due to the increased production of ROS-related proteins and ROS signaling-related transcription factors, such as ZAT10 and ZAT12.

### Ethanol enhances the detoxification of ROS under high-salinity stress condition

**Figure 3:**
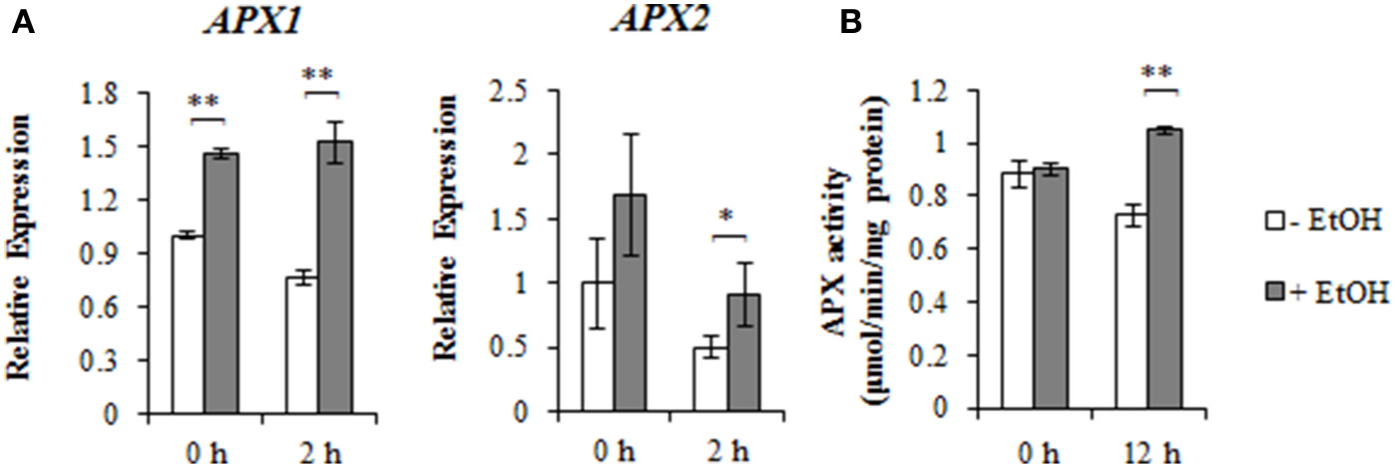
*AtAPX1* and *AtAPX2* expression and APX activity under salinity stress condition in the presence or absence of ethanol. **(A)** Relative *AtAPX1* and *AtAPX2* expression levels during a salinity stress treatment for 0 and 2 h in the presence or absence of 0.3% (51 mM) ethanol. The expression level of the unstressed plants treated with water was set as 1, and the *ACT2* gene was used as an internal standard. Each treatment was analyzed using 30 plants. Three biological repeats were performed. Error bars represent the mean ± *SD*. Significance was determined according to Student's *t*-test. ^*^P < 0.05; ^**^P < 0.001. **(B)** The APX activity during a 12 h salinity stress treatment in the presence or absence of 0.3% (51 mM) ethanol. Each treatment was analyzed using 30 plants. Three biological repeats were performed. Error bars represent the mean ± *SD*. Significance was determined according to Student's *t*-test. ^**^P < 0.001.

To characterize the molecular functions of ethanol treatments, the expression of the *ZAT10/12*-related ROS scavenger genes was analyzed by qRT-PCR. We confirmed that *AtAPX1* and *AtAPX2* expression levels increased in response to ethanol and NaCl treatments (Figure 3A). To clarify whether ethanol regulates cytosolic APX activity, an APX enzyme assay was performed. At 12 h after the NaCl treatment, total APX activity was higher in ethanol-treated plants than in untreated controls under the high-salinity stress condition (Figure 3B). In contrast, no significant differences were observed between ethanol-treated and untreated control plants under the non-stress condition. Our data indicated that ethanol induces the transcription of *APX2* and *APX1*, and enhances APX activity.

**Figure 4:**
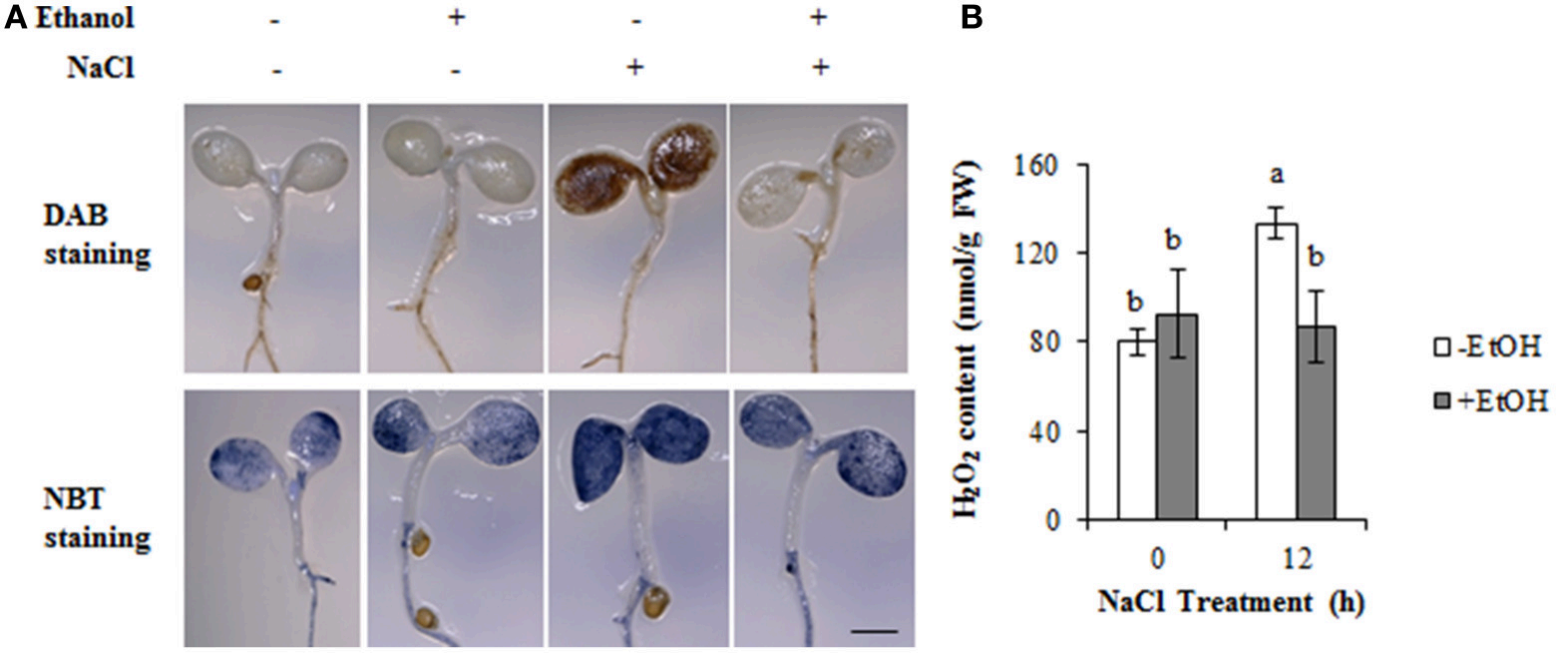
Accumulation of ROS in the cotyledon under high-salinity stress condition in the presence or absence of ethanol. **(A)** DAB and NBT staining was used to assess the accumulation of H_2_O_2_ and ${\text{O}}_{2}^{•-}$, respectively, in the cotyledons of *Arabidopsis thaliana* plants treated with NaCl for 12 h in the presence or absence of 0.3% (51 mM) ethanol. Bar = 1 mm. Each treatment was analyzed using 10 plants. Three biological repeats were performed. **(B)** H_2_O_2_ content in the cotyledons during a 12 h salinity stress treatment in the presence or absence of 0.3% (51 mM) ethanol. The experiments were conducted with three biological repeats. Error bars represent the mean ± *SD*. Statistical significance was determined by ANOVA, followed by *post-hoc* Tukey's tests. Means that differed significantly (*P* < 0.05) are indicated by different letters.

The accumulation of ${\text{O}}_{2}^{•-}$ and H_2_O_2_, which are the two main ROS components induced by salt stress, normally results in oxidative damage. We investigated the accumulation of ${\text{O}}_{2}^{•-}$ and H_2_O_2_ in ethanol-treated *A. thaliana* plants under salt stress conditions using NBT and DAB staining, respectively. The cotyledons of NaCl-treated plants were extensively stained by DAB, indicating H_2_O_2_ was highly accumulated under high-salinity stress condition. The ethanol treatment resulted in a lower accumulation of H_2_O_2_ (Figure 4A). The NBT staining results revealed slight differences among treatments, and the plants treated with ethanol accumulated less ${\text{O}}_{2}^{•-}$ than the control plants under the salt stress condition (Figure 4A).

The staining data were further confirmed by H_2_O_2_ content in shoots. The results were consistent with the DAB staining data. Under the control condition, there were no significant differences between ethanol-treated and non-treated plants (Figure 4B). However, after 12 h NaCl stress condition, the ethanol-treated plants could maintain the H_2_O_2_ level as stable as those in control condition, while the ethanol-nontreated plants showed higher concentration of H_2_O_2_ compared with the plants in the control condition and ethanol-treated plants under high-salinity stress condition (Figure 4B). These data indicate that ethanol enhances salinity stress tolerance by ROS detoxification in *A. thaliana*.

### Ethanol treatment enhances high-salinity tolerance by decreasing the accumulation of ROS in rice

**Figure 5:**
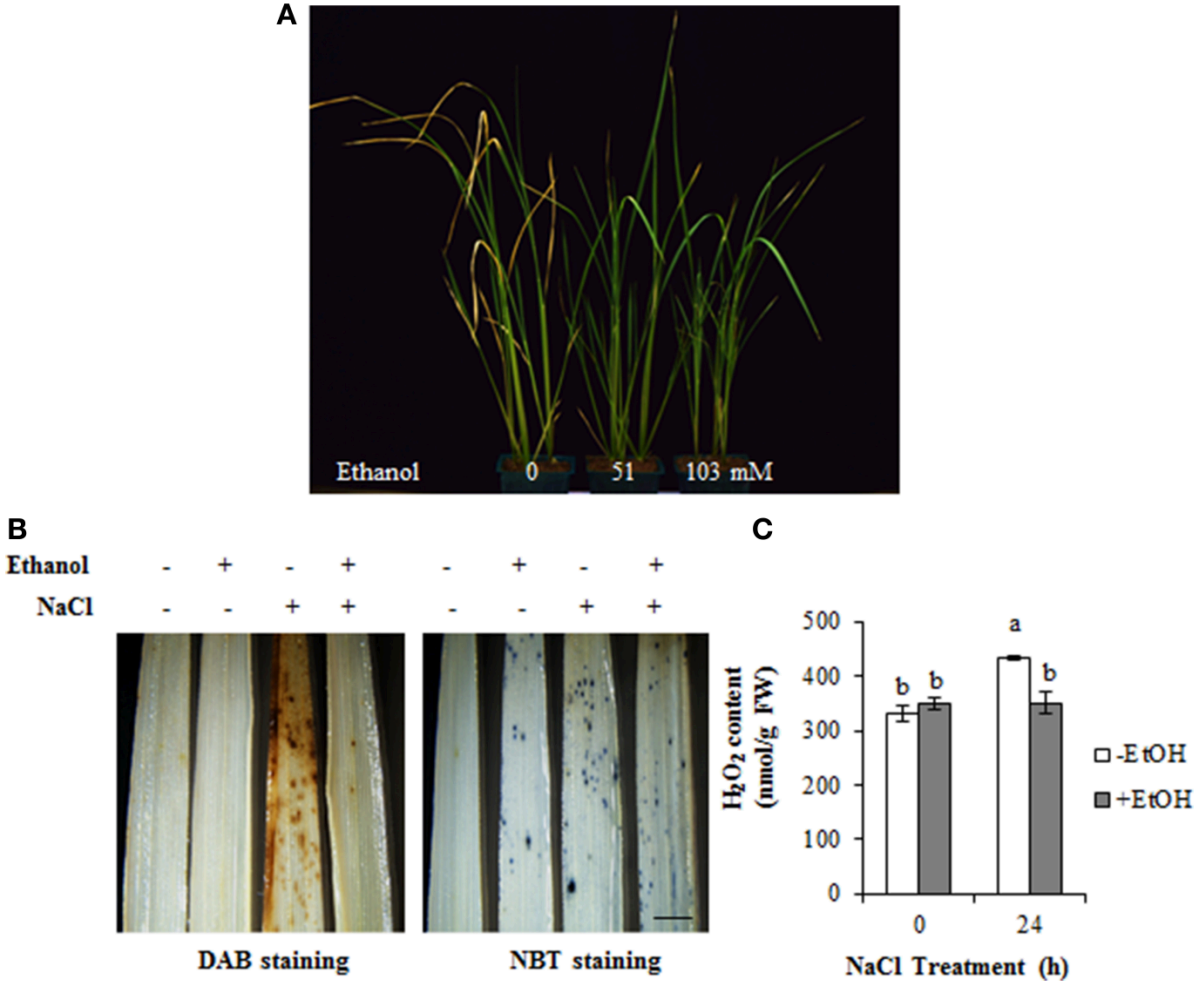
Ethanol enhances high-salinity stress tolerance in rice. **(A)** Phenotype of rice seedlings treated with 200 mM NaCl for 5 days in the presence or absence of 0, 0.3, and 0.6% ethanol (0, 51, and 103 mM, respectively). **(B)** DAB and NBT staining was used to assess the accumulation of H_2_O_2_ and ${\text{O}}_{2}^{•-}$ in the leaves of 14-day-old rice plants treated with 100 mM NaCl for 24 h in the presence or absence of 0.3% (51 mM) ethanol. Bar = 2 mm. **(C)** H_2_O_2_ content in the leaf extracts during a 24 h salinity stress treatment in the presence or absence of 0.3% (51 mM) ethanol. Statistical significance was determined by ANOVA, followed by *post-hoc* Tukey's tests. Means that differed significantly (*P* < 0.05) are indicated by different letters.

To confirm whether ethanol enhances the tolerance of monocots to salt stress condition, 14-day-old rice seedlings were treated with several ethanol concentrations. We observed that the leaves of untreated plants turned slightly yellow on 5 days after the NaCl treatment (Figure 5A). However, the leaves of salinity-stressed plants treated with 0.3 and 0.6% (51 and 103 mM, respectively) ethanol remained green, suggesting that ethanol enhances salinity stress tolerance in rice as well as *A. thaliana* (Figures 1, 5A).

Ethanol treatments enhanced ROS detoxification and improved the salt stress tolerance of *A. thaliana* plants. We used DAB and NBT staining to verify that salinity stress tolerance in rice is due to the detoxification of ROS. Rice leaves were more extensively stained by DAB under salinity stress condition than under control condition (Figure 5B). However, the intensity of the DAB staining decreased in ethanol-treated plants under salt stress condition, suggesting that ethanol inhibited ROS accumulation (Figure 5B). Additionally, there was no clear difference in the NBT staining of ethanol-treated and control plants under high-salinity condition (Figure 5B). The H_2_O_2_ content of rice leaves was measured and the data showed the highest concentration was detected in plants treated with NaCl for 24 h. In contrast, the plants treated with both NaCl and ethanol showed lower concentration of H_2_O_2_, which is similar with that of control condition (Figure 5C). These results confirmed our DAB staining data, implying that ethanol increases salinity tolerance in rice by inhibiting ROS accumulation, similar to its effects in *A. thaliana* plants.

## Discussion

Our findings indicated that ethanol enhances high-salinity stress tolerance in *A. thaliana* and rice by detoxifying ROS (Figures 1, 5). Microarray and qRT-PCR analyses of *A. thaliana* revealed that ethanol upregulates the expression of *AtZAT10, AtZAT12, AtAPX1*, and *AtAPX2* genes encoding transcription factors and the ROS scavenger under high-salinity condition (Figures 2B, 3A). Ethanol also increases APX activity in salt-stressed plants (Figure 3B), resulting in decreased H_2_O_2_ levels (Figures 4B, 5C).

In addition to APX, various H_2_O_2_-scavenging enzymes (e.g., CAT, GPX, GST, and PrxR) are reportedly involved in decreasing excess H_2_O_2_ generated under stress conditions (Mittler et al., 2004). Our microarray data indicated that the expression of several genes encoding H_2_O_2_-scavengers was upregulated by ethanol under high-salinity stress conditions (Figure 2A, Table 1), including *CAT2, GPX6* and *7, GSTs*, and *PrxR D*. These enzymes convert H_2_O_2_ to water to decrease excess H_2_O_2_ content (Mittler et al., 2004; Dixon, 2010). The upregulation of these ROS-scavenger–encoding genes may accelerate the decrease in toxic ROS content. However, during the conversion of H_2_O_2_ to water, glutathione, and ascorbate are used as enzyme co-substrates, and are recycled by GR, GLR, and DHAR (Mittler et al., 2004). The upregulated expression of *GR, GLR*, and *DHAR* may ensure there is a sufficient supply of ascorbate and glutathione for enzymatic reactions (Mittler et al., 2004). Glutathione-S-transferase catalyzes lipid hydroperoxides and prevents ROS-induced cell membrane damage (Dixon, 2010). Our microarray data confirmed that the expression of 20 *AtGST* genes is upregulated by ethanol (Table 1). Of these genes, *AtGSTU4* and *AtGSTU19* help mediate high-salinity stress tolerance (Sharma et al., 2014; Xu et al., 2015). The overexpression of *OsGSTU4* and *AtGSTU19* in transgenic *A. thaliana* improves salinity stress tolerance by inhibiting the accumulation of ROS and increasing GST activity (Sharma et al., 2014; Xu et al., 2015). A previous study concluded that GST activity in pumpkin plants is highly induced by 50 mM ethanol, which is equivalent to 0.3% ethanol (Fujita and Hossain, 2003). This finding supports our observation that ethanol upregulates the expression of *GST* genes (Table 1) to potentially increase the abundance of GST. These results suggest that the application of exogenous ethanol regulates the expression of genes encoding H_2_O_2_-scavenging enzymes and their related signaling proteins in salt-stressed *A. thaliana* plants.

The maintenance of the steady state between H_2_O_2_ and ${\text{O}}_{2}^{•-}$ levels, which is crucial for many molecular mechanisms in plant cells, is regulated by an appropriate balance between the associated scavenging activities (Miller et al., 2010). Our microarray data revealed that *AtAOX1A* expression is upregulated by ethanol under salinity stress condition (Table 1). In contrast, ethanol does not upregulate the expression of *AtSOD* genes, which encode enzymes responsible for catalyzing the dismutation of ${\text{O}}_{2}^{•-}$ to H_2_O_2_ and oxygen (Mittler et al., 2004). These observations are consistent with our NBT staining results, which indicated the differences in ${\text{O}}_{2}^{•-}$ content between ethanol-treated and untreated plants are minimal under salt stress condition (Figure 4A), and our DAB staining results, which revealed clear differences in H_2_O_2_ abundance (Figures 4A, 5B). Thus, ethanol influences the elimination of H_2_O_2_ rather than ${\text{O}}_{2}^{•-}$. The expression of several ROS-scavenging signaling genes is also upregulated by ethanol under salt stress condition (Table 1). The upregulation of the genes encoding ferritin and blue copper proteins, which prevent the formation of the highly toxic HO^•^ (Miller et al., 2010), might also contribute to ethanol-mediated ROS detoxification mechanisms.

In this study, the effects of five organic solvents have been tested (including ethanol, acetone, methanol, DMF, and DMSO). Among them, only ethanol enhanced the high-salinity stress tolerance (Figures 1A, B). This phenotype might be caused by a specific function of ethanol that leads to enhancement of high-salinity tolerance. Previous studies reported that spraying methanol and ethanol to tomato leaves enhanced plant growth under normal condition and that root applications of 5% ethanol and methanol caused severe plant damage (Rowe et al., 1994). It is expected that combination of application method and concentration of organic solvents has various effects on plant growth. Further experiments are necessary to analyze the detailed effect of organic solvents on plant growth.

The high-salinity stress tolerance test of *Arabidopsis* plants using the 24-well-plate system might cause hypoxia that leads to ethanol fermentation (Kato-Noguchi and Kugimiya, 2001). It raises a question whether high-salinity stress tolerance caused by ethanol is related to hypoxia or not. When we treated the rice seedlings grown on soil (not soaked rice seedlings) with ethanol for the salt stress test, ethanol enhanced salt stress tolerance (Figure 5A). These data showed that increased high-salinity tolerance by ethanol is independent of hypoxia effect.

We observed that the application of exogenous ethanol enhances rice tolerance to salt stress condition *via* the detoxification of H_2_O_2_ (Figure 5). In summary, the enhancement of high-salinity tolerance due to ethanol treatments might be conserved in dicot and monocot plants. Ethanol is a simple and inexpensive compound. Thus, it may be very useful for protecting important crops from high-salinity stress. Integrative omics-based studies may reveal additional factors affecting ethanol-mediated high-salinity stress tolerance.

## Author contributions

HN, KS, AM, and MS designed the study. HN, KS, AM, MM, CH, YS, MT, and YH conducted the experiments. HN, KS, and AM analyzed the data. HN, KS, AM, LT, YH, and MS reviewed the data and wrote the manuscript.

### Conflict of interest statement

The authors declare that the research was conducted in the absence of any commercial or financial relationships that could be construed as a potential conflict of interest.
