# Supplementary figures and images for: Ethanol Enhances High-Salinity Stress Tolerance by Detoxifying Reactive Oxygen Species in Arabidopsis thaliana and Rice
Source: Front Plant Sci. 2017 Jul 3;8:1001. doi: 10.3389/fpls.2017.01001 (PMC5494288; doi:10.3389/fpls.2017.01001)

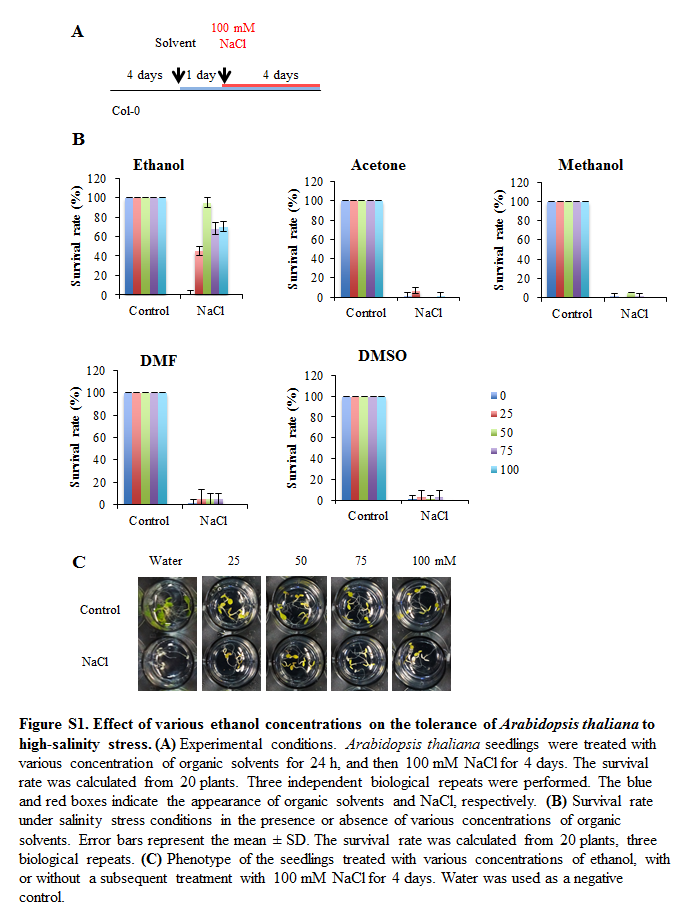

Supplement: Supplementary file 2 [file Image1.TIF]

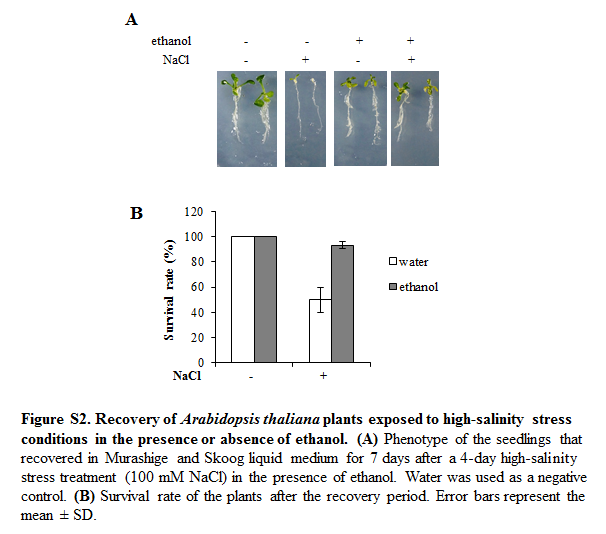

Supplement: Supplementary file 3 [file Image2.TIF]
